# Objectively Measured Sitting and Standing in Workers: Cross-Sectional Relationship with Autonomic Cardiac Modulation

**DOI:** 10.3390/ijerph16040650

**Published:** 2019-02-22

**Authors:** David M. Hallman, Niklas Krause, Magnus Thorsten Jensen, Nidhi Gupta, Marie Birk Jørgensen, Andreas Holtermann

**Affiliations:** 1 Centre for Musculoskeletal Research, Department of Occupational and Public Health Sciences, University of Gävle, Gävle 80637, Sweden; 2 Departments of Epidemiology and Environmental Health Sciences, Fielding School of Public Health, University of California Los Angeles, Los Angeles, CA 90095, USA; niklaskrause@ucla.edu; 3 Department of Cardiology, Copenhagen University Hospital, Herlev-Gentofte, 2900 Hellerup, Denmark; magnustjensen@gmail.com; 4 National Research Centre for the Working Environment, Copenhagen 2100, Denmark; ngu@nfa.dk (N.G.); marie.birk.joergensen@sund.ku.dk (M.B.J.); aho@nfa.dk (A.H.)

**Keywords:** age, accelerometer, cardiovascular disease, heart rate variability, occupational health, physical activity

## Abstract

Excessive sitting and standing are proposed risk factors for cardiovascular diseases (CVDs), possibly due to autonomic imbalance. This study examines the association of objectively measured sitting and standing with nocturnal autonomic cardiac modulation. The cross-sectional study examined 490 blue-collar workers in three Danish occupational sectors. Sitting and standing during work and leisure were assessed during 1–5 days using accelerometers. Heart rate (HR) and heart rate variability (HRV) were obtained during nocturnal sleep as markers of resting autonomic modulation. The associations of sitting and standing still (h/day) with HR and HRV were assessed with linear regression models, adjusted for age, gender, body mass index, smoking, and physical activity. More sitting time during leisure was associated with elevated HR (*p* = 0.02), and showed a trend towards reduced HRV. More standing time at work was associated with lower HR (*p* = 0.02), and with increased parasympathetic indices of HRV (root mean squared successive differences of R-R intervals *p* = 0.05; high-frequency power *p* = 0.07). These findings, while cross-sectional and restricted to blue-collar workers, suggest that sitting at leisure is detrimental to autonomic cardiac modulation, but standing at work is beneficial. However, the small effect size is likely insufficient to mitigate the previously shown detrimental effects of prolonged standing on CVD.

## 1. Introduction

Excessive leisure or total sitting time is associated with an elevated risk of cardiovascular disease (CVD) and all-cause mortality [1,2,3,4]. The causal neurobiological mediators of this relationship are however not established, although research suggests hemodynamic, inflammatory, and metabolic processes as the most likely mediators [5].

The autonomic nervous system is a key adaptive system involved in the regulation of various cardiovascular processes through the sympathetic and parasympathetic branches. Too much sitting may lead to imbalanced autonomic regulation in terms of increased sympathetic and reduced parasympathetic (vagal) activity [6,7,8], which could be detrimental to CVD due to elevated heart rate (HR) and blood pressure, reduced baroreflex sensitivity, and excess inflammation [8,9]. However, very few studies have probed the possible relationship of sitting time at work and leisure with autonomic activity [10], while research indicates that sufficient daily physical activity [11,12,13] and exercise [14] are associated with beneficial effects on parasympathetic activity. Thus, an investigation of the association between sitting time and autonomic activity is important for understanding potential mediating pathways, and for achieving specific exposure recommendations and successful prevention of CVD.

In contrast to sitting, the health effects of standing have received far less research attention. Overall, prolonged standing has been observed to be a risk factor of CVD morbidity and mortality [15,16,17,18]. A review from 2015 based on 11 studies suggests that prolonged standing at work is associated with CVDs such as varicose veins, increased blood pressure, and ischemic heart disease [16]. In a recent prospective study of 66,000 Japanese employees, those reporting to mostly stand at work were found to have a 20% increased risk for CVD mortality [15]. Another recent prospective study of a representative sample of >7000 Canadian adults reported that occupations involving predominantly standing work were associated with a doubled risk of developing heart disease compared to mostly sitting [18]. Yet another recent prospective cohort study of over 30,000 working U.S. women reported that those with pre-existing cardiovascular disease who were sitting and standing equally at their current job experienced a 2-fold increased risk of transitory ischemic attacks compared to those mostly sitting at work [19]. While posture-related hemodynamic changes may explain the increased risks from standing postures [17], other mechanisms such as changes in autonomic activity due to discomfort and pain [20] associated with prolonged standing [21] may also be involved. Investigating the association between standing time and autonomic activity may provide further insight into this relationship.

Overall, few observational studies have relied on accurate and precise technical measurements of sitting and standing positions to determine associations with CVD, including autonomic cardiac modulation. Self-reported measurements of sitting and standing are prone to inaccuracy and bias, which can lead to distorted associations with cardiovascular health outcomes [22,23,24,25]. Thus, objective measurements of sitting and standing are crucial to obtain trustworthy results, while also allowing a detailed classification of different postures and activity types. For instance, differentiating standing still from standing postures including lower limb physical activity is particularly important as these activities result in different physiological effects [26].

Further, as the occurrence, contextual correlates, and potential for behavioral change of sitting and standing depend on the domain, such as work and leisure (i.e., non-working hours while awake), it is important to distinguish exposure at work and leisure when examining associations with health outcomes. Epidemiological studies indicate that leisure-time physical activity is beneficial for cardiovascular health, while occupational physical activity can be even detrimental [27,28,29,30]. This may be explained by the constrained and persistent nature of physical activity at work, and insufficient recovery resulting in sustained elevations in heart rate and blood pressure [28].

HR is a general marker of cardiorespiratory fitness and autonomic cardiac activity, showing high predictive validity for CVD outcomes [31,32,33,34], particularly if determined during stable resting conditions, including sleep [35]. Heart rate variability (HRV) is an established indicator of intrinsic autonomic cardiac modulation, reflecting mainly parasympathetic influences, but also sympathetic and baroreceptor modulations [36]. Reduced HRV at rest or over 24 h generally indicates aberrant autonomic regulation, and is associated with an increased risk and a poor prognosis of CVD [37,38,39]. HRV can preferably be determined during nighttime sleep to minimize acute effects of daily physical activity and mental stress [40]. Thus, assessing nocturnal resting HR and HRV can provide insight into the long-term effects of sitting and standing on autonomic regulation of the cardiovascular system.

The aim of this study is to examine the cross-sectional association between objectively measured sitting and standing (at work and leisure) with nocturnal resting autonomic modulation in a sample of blue-collar workers.

## 2. Materials and Methods

### 2.1. Design and Study Population

This is a cross-sectional analysis of data from the Danish PHysical ACTivity cohort with Objective Measurements (DPHACTO) collected between spring 2012 and spring 2013. The study protocol of DPHACTO is described elsewhere [41].

The study population consisted of blue-collar workers recruited from 15 Danish workplaces across three industrial sectors (cleaning, manufacturing and transportation). The companies were chosen to obtain a rather homogenous population in terms of socioeconomic status and with a sufficient dispersion in occupational physical activities, including sitting and standing [42,43].

Inclusion criteria for participation were employment within any of the selected workplaces, permission from the company to perform measurements during paid working hours, holding a blue-collar job, and taking part in the objective measurements of physical activity and HR. Exclusion criteria were white-collar work, managing position, student/trainee, pregnancy, or allergy to adhesives. Workers without valid data (see criteria below) on objective measurements were also excluded.

The flow of participants was previously reported [13]. In total, 2107 employees were invited to participate and 1119 consented. After excluding white-collar workers (*n* = 186), managers (*n* = 17), student/trainees (*n* = 13), and pregnant women (*n* = 2), 901 blue-collar workers were eligible. Of them, 755 participated in objective measurements of physical activity and HR, with 514 providing valid records. Of them, 490 provided questionnaire data on potential confounding factors. Thus, the final sample included in the analysis consisted of 490 workers (54% of the eligible workers).

All participants provided their written informed consent prior to participation. The study was conducted according to the Helsinki Declaration, approved by the Danish data protection agency, and evaluated by the Regional Scientific Ethics Committee of Copenhagen, Denmark (H-2-2012-011).

### 2.2. Data Collection

Data collection consisted of a brief web-based questionnaire, a health check with a physical examination, and ambulatory measurements of physical activity and HR [41]. During the ambulatory recordings, the participants were instructed to wear the technical equipment (accelerometers and a HR monitor) for 24 h a day during 4–5 consecutive days, and to perform a reference measurement in an upright standing position without any movement for 15 s each day to optimize activity detection from the accelerometer signals. They were also instructed to remove the equipment if it caused any kind of discomfort. A diary was used each day of the recording to note time-stamps (in minutes resolution) of working hours, leisure time (i.e., non-working hours excluding bedtime), bedtime and reference measurements. The equipment was returned to the research staff after testing.

### 2.3. Assessment of Sitting and Standing

Sitting and standing were measured using two tri-axial accelerometers (Actigraph GT3X+, ActiGraph LLC, Pensacola, FL, USA) attached to the thigh and trunk using double-sided tape. Detailed descriptions of the accelerometers, data processing, and classification of activity types are found elsewhere [10,43,44,45].

The accelerometer recordings were initialized using the accompanying software Actilife (ActiGraph LLC, Pensacola, FL, USA), while the data obtained from the accelerometer records were processed and analyzed off-line using the custom-made software Acti4 (The National Research Centre for the Working Environment, Copenhagen, Denmark; and The Federal Institute for Occupational Safety and Health, Berlin, Germany). This software determines periods of lying, sitting, standing, and physical activity (walking, climbing stairs, running and cycling) with high sensitivity and specificity [44,45,46]. Standing and physical activity was assessed using only the thigh accelerometer, as previously described [44,45], while data from the thigh and trunk accelerometers were combined to distinguish lying from sitting. We assessed only standing still—that is, standing with minimal lower limb physical activity but not including occasional stepping or walking—which we operationalized as periods when the maximum standard deviation of the thigh accelerometer was below 0.1 G for all three axes [44].

Non-wear time for each accelerometer was determined using an automatic algorithm [47] (i.e., detection of periods >90 min with 0 accelerometer counts) combined with diary reports (i.e., self-reported non-wear time) and visual inspection of the data (i.e., detection of artefacts or missing data).

Valid accelerometer records had to contain at least one complete day with valid data at work and leisure. The criteria for valid work and leisure periods were ≥4 h per day of accelerometer wear-time, or 75% of the average wear-time across days for the individual [48].

The time-stamps obtained from the diary were merged with the accelerometer records using the Acti4 software to determine sitting and standing times during work and leisure time, respectively. Sitting and standing time was calculated for work and leisure for each measurement day and averaged across days (h/day), and used as continuous variables in the statistical analyses.

### 2.4. Assessment of Heart Rate Variability During Sleep

A heart rate monitor (Actiheart, CamNtech Ltd., Cambridge, UK) was used during 24 h across 1–5 days to sample R-R intervals (RRI) from the electrocardiogram at 1000 Hz resolution. The device was attached using a two-lead configuration with pre-gelled Ag/AgC1 electrodes on cleansed skin. The RRI time-series were processed off-line for automatic removal of abnormal RRI and technical artefacts. RRI records were excluded if they contained less than four hours of data with sufficient quality during sleep [13].

Data processing and analysis of HRV were done according to previously described procedures [10,49]. HRV indices were obtained from the RRI time series in both the time domain (root mean squared successive differences of RRI (RMSSD); standard deviation of RRI (SDNN)) and the frequency domain (low-frequency power (LF) 0.04 to 0.15 Hz; high-frequency power (HF) 0.15 to 0.4 Hz) based on non-overlapping 5-minute windows [36]. A robust period detection procedure was used to determine the HRV spectral power components [49].

RMSSD and HF are indicators of parasympathetic (vagal) activity, LF reflects both sympathetic and parasympathetic cardiac modulations, and SDNN reflects overall HRV [36].

Diary information of time in bed combined with accelerometer measurements were used to determine nocturnal periods without movement, classified as sleep [10]. For each sleep interval, the three 5-min intervals with the lowest HR were determined, whereby the mean HR and HRV values were calculated and averaged across nights. Thus, HR and HRV were obtained during the absolute resting state (lowest HR) to reduce potential influences of nocturnal variations in sleep quality on autonomic cardiac modulation. Non-normal distributions were found for RMSSD, LF, and HF and were transformed using the natural logarithm (ln).

### 2.5. Assessment of Possible Confounders and Effect Modifiers

Possible confounding factors and effect modifiers were selected a priori based on theory and empirical data of their association with both exposure and outcome. The study included only skilled (43%) and unskilled (57%) blue-collar workers to minimize possible socioeconomic confounding.

Age (years) and gender (“male” or “female”) were determined using the civil registration number. The questionnaire was used to obtain self-reported information about smoking: “yes” (daily smoking) or “no” (occasionally, formerly or never smoked); seniority in the current job (years); current use of CVD drugs (“yes” or “no”) by two items about anti-hypertensive and heart/lung disease medications; and one item each for usage of antidepressants, analgesics, and other medication during the past three months.

Body mass index (BMI, kg/m^2^) was calculated from objectively measured weight (Tanita, model BC418 MA) and height (Seca, model 123).

Time in moderate-to-vigorous physical activity (MVPA) during leisure time, was assessed by accelerometers (see above), and calculated as the total time of brisk walking, climbing stairs, running, and cycling in h/day [13].

Resting blood pressure (mmHg) was assessed after 15 min of seated rest using the Omron M6 Comfort (Omron health Care, Netherlands) on the upper left arm. Systolic and diastolic blood pressure was determined by averaging three readings, whereby a systolic blood pressure ≥140 mm/Hg or a diastolic blood pressure ≥90 mmHg indicated hypertension [50].

### 2.6. Statistical Analyses

Statistical analyses were conducted using SPSS version 24 (IBM, Armonk, NY, USA)). Multiple linear regression analyses with point estimates (B) and 95% confidence intervals (CIs) were used to determine the association between exposure (sitting and standing time at work and leisure expressed in h/day) and nocturnal HR and HRV indices, without adjustment for potential confounders (Model 1). Sitting and standing time were entered in separate models that included both work and leisure. The same regression models were also constructed with adjustment for age, gender, BMI, smoking, and MVPA during leisure (Model 2). Adjusted models were also constructed with additional adjustment for MVPA at work.

Secondary models were constructed as above to examine possible effect modification by adding multiplicative interaction terms (sitting or standing exposure × modifier) for age (<45 or ≥45 years; close to the mean age of this occupational study sample), and CVD, i.e., having hypertension and/or reporting CVD medication (hypertensives and heart/lung disease). Interaction effects with *p*-values < 0.1 were further probed by stratified analyses and scatter plots of the linear association in each stratum. In addition, multiplicative interaction effects were examined between exposure at work and leisure.

Linearity was examined by plotting mean values of HR and HRV indices with 95% CI across quartiles of sitting and standing at work and leisure. There were no marked deviations from linear relationships. Multicollinearity among the independent variables was examined by inspection of the variance inflation index and tolerance values obtained by collinearity diagnostics. Multicollinearity was not an issue in any model. Also, the residuals showed close to normal distributions and homoscedasticity for all models (i.e., HR, SDNN, and ln-transformed RMSSD, LF, and HF).

## 3. Results

Characteristics of the total study population are shown in Table 1 and data stratified on age are shown in Table 2. Workers ≥45 years of age were similar to those <45 years in most characteristics, apart from medication use and hypertension being more prevalent among older workers. Valid accelerometer recordings were obtained for 2.7 days on average, including 7.6 h/day at work and 8.8 h/day during leisure. The exposure to sitting (population mean: 2.4 h at work and 4.8 h during leisure) and standing (population mean: 2.5 h at work and 1.7 h during leisure) showed a substantial dispersion between workers, regardless of age (Table 1 and Table 2). Descriptive data on nocturnal resting HR and HRV are shown in supplement (Table S1).

### 3.1. Sitting Time and Nocturnal Resting Heart Rate and Heart Rate Variability

At work, sitting time showed no association with nocturnal resting HR and non-significant inverse associations with overall (SDNN) and parasympathetic measures of HRV (ln RMSSD and ln HF) in adjusted models (Table 3). During leisure, each hour of sitting per day was associated with an increase of 0.6 bpm in HR in the adjusted model (Table 3), which persisted after additional adjustment for MVPA at work (B = 0.6; 95% CI 0.1 to 1.1). More sitting time during leisure was associated with reduced HRV across all measures (SDNN, ln RMSSD, ln HF, and ln LF) in the crude model, but effects were reduced by about 40‒88% and no longer statistically significant (*p* > 0.05) after adjustment for confounders.

No statistically significant interaction effect was observed between sitting time at work and leisure on HR (*p* = 0.11) and HRV (all *p* > 0.60).

There was no statistically significant interaction between sitting time and age (work: SDNN *p* = 0.10, ln LF *p* = 0.12, all other *p* > 0.40; leisure: all *p* > 0.40). Sitting during leisure showed a statistically significant (*p* = 0.08) interaction with CVD on HR (B = 0.9; 95% CI −0.1 to 1.9), but not HRV (all *p* > 0.40); reflecting a stronger positive association in workers with CVD (B = 1.0; 95% CI 0.2 to 1.8) than without CVD (B = 0.2; 95%CI −0.4 to 0.9).

### 3.2. Standing Time, Nocturnal Resting Heart Rate, and Heart Rate Variability

At work, each hour of standing per day was associated with a 0.7 bpm reduction in nocturnal resting HR in the adjusted model (Table 4). More standing time at work was associated with increased nocturnal HRV; being close to statistically significant in the adjusted model for parasympathetic HRV indices (ln RMSSD *p* = 0.05; ln HF *p* = 0.07). Similar estimates and *p*-values were found after additional adjustment for MVPA at work (results not shown).

During leisure, each hour of standing per day was associated with a 0.5 bpm elevation in HR in the adjusted model, but this association was not statistically significant (Table 4). More standing time during leisure was associated with increased nocturnal HRV, but only ln LF was close to being statistically significant (*p* = 0.08). There was no statistically significant interaction between standing time at work and leisure on HR (*p* = 0.23), SDNN (*p* = 0.60), ln RMSSD (*p* = 0.89), ln HF (*p* = 0.90), and ln LF (*p* = 0.22).

Statistically significant interactions were observed for standing during leisure with age (SDNN, *p* = 0.08; ln RMSSD, *p* = 0.05; ln HF, *p* = 0.08) and CVD (ln RMSSD, *p* = 0.04; ln HF, *p* = 0.06).

**Figure 1:**
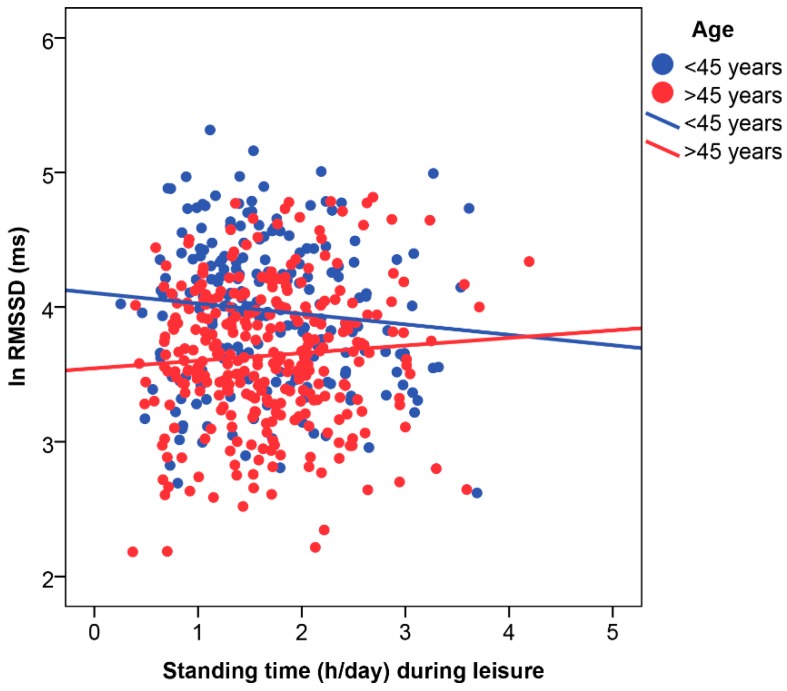
Interaction between age and standing time during leisure on nocturnal parasympathetic cardiac modulation. RMSSD: root mean square of successive differences between R-R intervals; ln natural: logarithm.

Specifically, age interacted with standing during leisure for overall HRV; i.e., SDNN (B = 5.0; 95% CI −0.6 to 10.7), and parasympathetic indicators (Figure 1); i.e., ln RMSSD (B = 0.1; 95% CI 0.0 to 0.3) and ln HF (B = 0.3; 95%CI −0.0 to 0.5). These interactions indicated that more standing time was associated with reduced nocturnal HRV in younger (<45 years) workers, but increased HRV in older workers (≥45 years). Results stratified by age are shown in supplement (Table S2).

**Figure 2:**
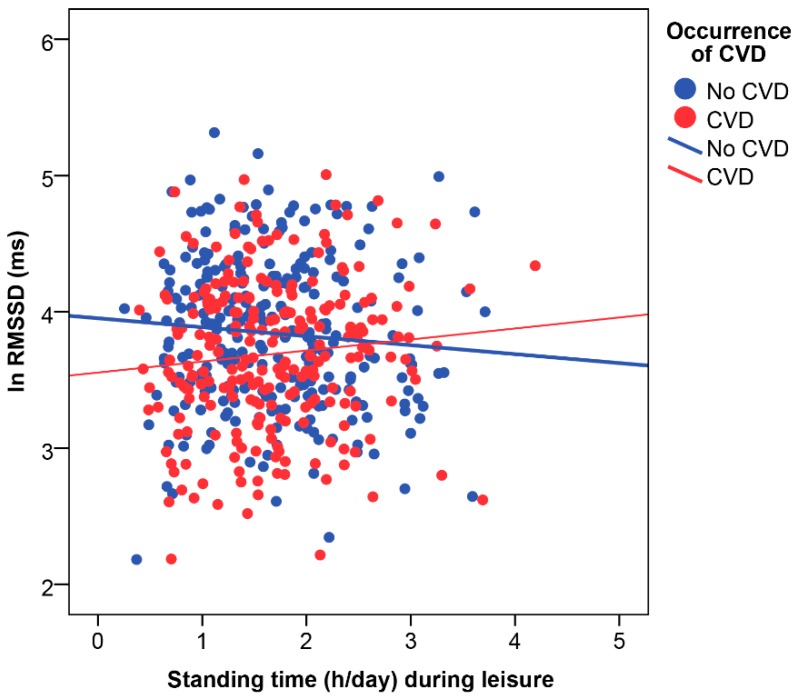
Interaction between cardiovascular disease (CVD) and standing time during leisure on nocturnal parasympathetic cardiac modulation. RMSSD: root mean square of successive differences between R-R intervals; ln: natural logarithm.

Occurrence of CVD interacted with standing during leisure for parasympathetic HRV indices (Figure 2); i.e., ln RMSSD (B = 0.2; 95% CI 0.0 to 0.3) and ln HF (B = 0.3; 95% CI −0.0 to 0.6). These interactions indicated that more standing during leisure was associated with reduced nocturnal HRV in those without CVD, but increased HRV in those with CVD. Results stratified by CVD are shown in supplement (Table S3). There was no statistically significant interaction between CVD and standing at work (*p* > 0.10).

## 4. Discussion

To our knowledge, this is the first study investigating the association between objectively measured sitting and standing (evaluated separately for work and leisure) with resting HR and HRV during nocturnal sleep, as markers of intrinsic autonomic activity. We found that sitting time was associated with elevated HR, but only for sitting during leisure. Standing time was associated with lower HR, and higher HRV, implying enhanced parasympathetic activity, but only for standing at work. For standing during leisure, effects were moderated by age and prevalent CVD.

### 4.1. Sitting Time and Nocturnal Autonomic Modulation

Each hour of sitting during leisure time was associated with an elevation in nocturnal resting HR of 0.6 beats per minute in the adjusted model (Table 3), which could indicate an elevated risk for CVD and all-cause mortality [32,35,51]. Thus, the observed effect is of potential relevance for cardiovascular health in blue-collar workers.

We found negative associations between sitting during leisure and parasympathetic indices and overall HRV in the crude model, although the effects were reduced and no longer statistically significant after adjustment for confounders (Table 3). This suggests that sitting more during leisure has a detrimental effect on resting HR, partly due to other mechanisms than attenuated parasympathetic cardiac modulation. Still, the adjusted negative association between sitting during leisure and HRV was close to statistically significant for the LF component of HRV, which reflects a mix of baroreceptor, sympathetic and parasympathetic modulations of the heart [52]. However, as this effect was relatively small, the clinical relevance can be questioned.

We are only aware of one previous study on sitting time and HRV, which showed no effect of sitting during leisure on nocturnal autonomic modulation among workers, while more sitting at work was associated with autonomic imbalance, i.e., reduced HRV [10]. However, we could not confirm this finding in the current sample of blue-collar workers. The marginal associations for sitting during work with nocturnal HR and HRV in the current population of workers may be explained by the occurrence of other work exposures during periods of non-sitting. As blue-collar work often contains high physical work demands known to be associated with poor cardiovascular health [27], sitting more during leisure may reflect the need for recovery from high work demands of these workers. However, we could not find a positive association between MVPA at work and sitting during leisure (*r* = −0.1), which suggests that the trend for a decrease in HRV associated with more sitting during leisure time cannot be explained by increasing physical work demands. This is in line with a recent study by Wang et al. 2016 that did not find any evidence for the mediation by leisure-time physical activity of an observed increased morality risk associated with occupational physical activity [53].

In summary, the results are consistent with previous epidemiological studies of a detrimental effect of excessive sitting time during leisure on cardiovascular health [1,2,3,30]. There is only limited support for autonomic imbalance as a physiologic pathway between sitting and cardiovascular health.

### 4.2. Standing Time and Nocturnal Autonomic Modulation

In contrast to sitting, we found that each hour of standing during work was associated with a 0.7 bpm reduction in nocturnal resting HR in the adjusted model (Table 4). Based on other epidemiological studies, this change in HR could indicate a reduced risk for CVD and all-cause mortality [32,35,51]. More standing during work was also associated with increases in parasympathetic measures of HRV, as indexed by RMSSD and HF. Although these effects were small and borderline statistically significant, they suggest that more standing time during work may be associated with enhanced parasympathetic activity during sleep in blue-collar workers.

Clearly these observations cannot explain the detrimental effects of prolonged standing at work on cardiovascular health reported in epidemiological studies [15,16,17,18]. Those effects are thought to be mediated at least in part by increases in HR and blood pressure according the established hemodynamic theory of atherosclerosis [17,54], which is in alignment with the consistent positive association of HR and blood pressure with CVD and mortality in epidemiological studies and confirmed in animal experiments and pharmacological randomized controlled trials [32,51,55]. A possible explanation for these apparently contrasting results is the use of different methods for assessing standing exposure. We used objective measurements (accelerometers) of standing, which allowed us to separate the effects of time in standing still from standing with occasional or sustained periods of lower limb physical activity. Previous epidemiological studies on standing at work and CVD have relied on expert rating or self-reported measures of exposure without clearly differentiating standing still from more dynamic forms of physical activity while standing. Thus, their observed effects may relate to a combination of a standing work posture with associated unmeasured physical activities with higher cardio metabolic demands such as walking. However, one study predicting 11-year progression of atherosclerosis by self-reported standing did control for self-perceived heaviness of work, thereby reducing the influence of other physical activities on the outcome [17]. Furthermore, our study reflects the independent effect of an average of 2.5 hours of standing still at work and not the effects of a predominantly standing work posture that has recently been shown to double the risk of incident heart disease [18].

Our finding of a cross-sectional association between standing still at work and nocturnal resting HR and HRV needs confirmation in longitudinal studies. Any potential beneficial health effects of standing via HRV would also need verification in future prospective epidemiologic studies. Future studies should also explore non-linear dose–response relationships and threshold effects for different types of standing and other upright work postures with different degrees of ambulation and upper body activities [26]. It is important to note that our study did not investigate any chronic cardiovascular health effects and that our findings do not rule out detrimental effects of standing on chronic CVD outcomes via other pathways.

Consequently, although this study indicates beneficial effects of standing at work on nocturnal autonomic modulation, it does not provide any evidence for beneficial effects of standing at work on CVD risk and does not support a recommendation of more standing at work.

The lack of any association of standing time during leisure with HR reduction or HRV increase does not support any mediating role of nocturnal autonomic modulation for cardiovascular health effects associated with standing postures. Finally, any results must be interpreted with extra caution due to the possibility of a healthy worker effect, i.e., workers who developed musculoskeletal or cardiovascular symptoms or diseases due to prolonged standing that led to increases in HR or decreases in HRV might have changed to jobs with less standing exposure, resulting in biased results with enhanced nocturnal modulation capacity among those remaining exposed to more standing time at work.

In summary, more standing time at work was associated with lower nocturnal resting HR and possibly increased parasympathetic measures of HRV, which are beneficial effects on autonomic regulation during nighttime that may translate into reduced CVD risk based on the known association of HR and HRV with CVD. However, even if confirmed in other studies, such marginal improvements in nocturnal resting autonomic regulation (i.e., reduction of 0.7 beats per minute in resting HR) may be insufficient to counteract the detrimental cardiovascular effect of a disproportionally higher HR (i.e., 5–10 beats per minute in working HR due to the standing posture alone) and concomitant blood pressure increases during daytime standing work on CVD risk, as documented in the literature cited above.

### 4.3. Effect Modification by Age, Cardiovascular Disease Status, and Physical Activity Domain

The observed interaction between sitting during leisure and CVD status on nocturnal resting HR is suggestive of a larger detrimental cardiovascular effect of sitting in workers with CVD than in those without CVD. Thus, reducing sitting time during leisure may be particularly important for workers with CVD.

We found no statistically significant association for standing during leisure in the whole sample, but interaction effects were observed for several HRV indices with age and occurrence of CVD. Stratified analyses indicated positive associations with HRV mostly among workers aged above 45 years and in workers with CVD (Tables S2 and S3). For instance, each one hour increment in standing during leisure was associated with an approximately 5% increase in SDNN (i.e., overall HRV) among workers with CVD. A meta-analysis including eight studies (*n* = 21,988) of HRV and CVD morbidity and mortality found that each 1% increase in SDNN was associated with a 1% risk reduction of CVD morbidity and mortality [39]. While this suggests that the effect of standing on nocturnal autonomic modulation and cardiovascular health depends on age and CVD, the underlying mechanisms are unclear and deserve further investigation. One possible mechanism is that standing postures require a wide range of HR to meet oxygen demands [56]. Thus, activation of HR modulation mechanisms during standing may counteract the potential reduction of that ability due to age- or disease-related declines in physical activity or fitness [57].

Observed effects of body posture differed by physical activity domain. Sitting during leisure had a detrimental effect on nocturnal autonomic modulation, while sitting at work had no effect, and standing at work had a beneficial effect while standing during leisure had no effect. These observations are consistent with the strong interactions between occupational and leisure-time physical activity in relationship to HRV as reported earlier from this study population [13] and constitute another manifestation of the so-called physical activity health paradox repeatedly described in the literature comparing the effects of occupational and leisure-time physical activity [27,28,58,59,60]. These observations further support the need to disentangle these effects in future research on the health effects of physical activity [28,29].

### 4.4. Study Strengths and Limitations

The objective measurements of sitting and standing by multiple accelerometers in a relatively large occupational sample are an obvious strength of this study. More accurate and precise assessments of exposure minimize the risk of underestimating any health effects [22]. As we measured both occupational and leisure time physical activity objectively, potential confounding by MVPA could be controlled in multivariate regression analyses. Determining resting HR and HRV during sleep also minimized the acute effects of movement and change in body posture on HRV occurring during day time. Further, we accounted for several other relevant factors (e.g., age, gender, BMI, current smoking, and CVD) potentially influencing physical behaviors and autonomic regulation. Still, residual confounding by non-measured factors cannot be ruled out. For instance, we did not assess psychological stress, which could have influenced HRV during the sleep period [61]. Also, HRV indices are mainly determined by parasympathetic cardiac modulation [52], and therefore this study provides limited information on the relationship of sitting and standing with sympathetic cardiac activity. Another limitation of the study is the cross-sectional design limiting causal inferences. We do not know if work postures caused observed autonomic regulation differences or vice versa. As noted above, workers suffering from impairment of autonomic regulation or related cardiovascular diseases may select into jobs with work postures that do not further reduce their capacity for autonomic regulation. Our results are limited to blue-collar workers and cannot be generalized to other occupational groups, such as office workers, that differ materially in the type and intensity of physical activities and other work exposures while standing or sitting. Overall, the observed effect sizes were small and the marginally significant *p*-values must be interpreted cautiously due to the risk of type 2 errors. Finally, we addressed sitting and standing in separate models adjusted for MVPA. However, as time use in different physical behaviors is co-dependent and compositional, further research should investigate the association of the whole composition of behaviors with cardiac autonomic modulation, e.g., using compositional data analysis [62], while considering that the health effects of similar activities may differ between work and leisure domains.

## 5. Conclusions

We found that more sitting time during leisure was associated with a potentially harmful elevation in nocturnal resting HR, possibly due to other mechanisms than autonomic imbalance. In contrast, more standing time during work was associated with a reduction in nocturnal resting HR and increases in parasympathetic measures of HRV, which indicates a potentially beneficial effect on cardiac autonomic modulation. These findings, although cross-sectional and restricted to blue-collar workers, support a recommendation to reduce sitting time during leisure. Although we found a beneficial effect of standing time at work on autonomic cardiac modulation, we cannot preclude a potential harmful effect of standing on CVD that may occur via much bigger increases in ambulatory heart rate during standing work that more than outweigh the small reduction at night, and due to other pathways. Thus, the results of this study alone do not justify recommendations to increase the amount of standing in blue-collar workers.

Further studies need to evaluate the effects of sitting and standing during work and leisure on cardiovascular health for different occupational groups characterized not only by the amount spent in certain postures but also by type and intensity of concomitant other activities and exposures. Specifically, we recommend using objective measurements of exposure to distinguish the effects of sitting, standing still, upright posture, and physical activity on CVD risk. Future studies on the pathophysiological pathways between body posture and CVD outcomes should use a longitudinal design and assess HR and HRV simultaneously with ambulatory blood pressure to determine the independent and combined effects of different potential hemodynamic and autonomic regulation pathways.

## Figures and Tables

**Table 1:** Characteristics of the study population (*N* = 490).

Variable	*N*	*n*	%	Mean (SD)	Range
Age (years)	490			45 (10)	18–68
Gender (female)	490	215	43.9		
BMI (kg/m^2^)	490			27.4 (4.7)	18.1–43.8
Smoking (yes)	490	121	24.7		
Sector	490				
Cleaning		99	20.2		
Manufacturing		349	71.2		
Transportation		42	8.6		
Seniority (years)	479			13.8 (10.3)	0–45
MVPA leisure (h/day)				0.7 (0.4)	0.1–2.0
Medication	489				
CVD medication ^a^		85	17.4		
Antidepressants ^b^		15	3.1		
Analgesics ^b^		93	19.0		
Other medication ^b^		114	23.3		
Hypertension ^c^		188	38.4		
Accelerometer wear-time	490				
Number of measured days				2.7 (1.0)	1–5
Work hours (h/day)				7.6 (1.2)	4–14
Leisure time (h/day)				8.8 (1.5)	4–13
Exposure at work	490				
Sitting (h/day)				2.4 (1.7)	0.2–9.2
Standing (h/day)				2.5 (1.1)	0.2–6.7
Exposure during leisure	490				
Sitting (h/day)				4.8 (1.3)	1.8–10.2
Standing (h/day)				1.7 (0.7)	0.3–4.2

^a^ Current use of anti-hypertensive or heart/lung disease medications. ^b^ Use of antidepressants, analgesics, and other medication during past three months. ^c^ Hypertension: systolic blood pressure ≥140 mm/Hg or diastolic blood pressure ≥90 mm/Hg. MVPA: moderate-to-vigorous physical activity; BMI: body mass index; CVD: cardiovascular disease.

**Table 2:** Characteristics of the study population stratified by age groups.

Variable		Age < 45 years (*N* = 196)					Age ≥ 45 years (*N* = 294)			
	*N*	*n*	%	Mean (SD)	Range	*N*	*n*	%	Mean (SD)	Range
Age (years)	196			36 (7)	18–44	294			52 (5)	45–68
Gender (female)	196	76	38.8			294	139	47.3		
BMI (kg/m^2^)	196			26.8 (4.8)	18.4–43.8	294			27.8 (4.7)	18.1–43.5
Smoking (yes)	196	46	23.5			294	75	25.5		
Sector	196					294				
Cleaning		35	17.9				64	21.8		
Manufacturing		145	74.0				204	69.4		
Transportation		16	8.2				26	8.8		
Seniority (years)	192			9.6 (6.9)	0.1–29.2	287			16.5 (11.3)	0.2–45.0
MVPA leisure (h/day)	196			0.7 (0.3)	0.1–1.9	294			0.7 (0.4)	0.1–2.0
Medication	195					294				
CVD medication ^a^		13	6.7				72	24.5		
Antidepressants ^b^		10	5.1				5	1.7		
Analgesics ^b^		34	17.4				59	20.1		
Other medication ^b^		32	16.4				82	27.9		
Hypertension ^c^	196	52	26.5			294	136	46.3		
Accelerometer wear-time										
Number of measured days	196			2.6 (1.0)	1–5	294			2.8 (0.9)	1–5
Work hours (h/day)	196			7.6 (1.3)	4.5–12.0	294			7.6 (1.2)	3.8–13.7
Leisure time (h/day)	196			8.6 (1.5)	4.2–13.1	294			9.0 (1.5)	4.5–13.4
Exposure at work										
Sitting (h/day)	196			2.5 (1.6)	0.4–9.2	294			2.3 (1.7)	0.2–8.0
Standing (h/day)	196			2.5 (1.2)	0.2–6.3	294			2.5 (1.1)	0.2–6.7
Exposure during leisure										
Sitting (h/day)	196			4.5 (1.2)	1.8–8.0	294			5.0 (1.4)	1.9–10.2
Standing (h/day)	196			1.7 (0.7)	0.3–0.7	294			1.7 (0.7)	0.4–4.2

^a^ Current use of anti-hypertensive or heart/lung disease medications. ^b^ Use of antidepressants, analgesics, and other medication during past three months. ^c^ Hypertension: systolic blood pressure ≥140 mm/Hg or diastolic blood pressure ≥90 mm/Hg. MVPA: moderate to vigorous physical activity; BMI: body mass index; CVD: cardiovascular disease.

**Table 3:** Associations between sitting time (h/day) and nocturnal resting heart rate and heart rate variability by activity domain. Results from linear regression analyses. Danish Physical activity cohort with objective measurements (DPHACTO), 2013.

	Sitting at Work			Sitting During Leisure		
	B	95% CI	*p*	B	95% CI	*p*
Unadjusted model (*n* = 490)						
Heart rate (bpm)	−0.19	−0.59 to 0.21	0.35	0.83	0.33 to 1.34	<0.001
SDNN (ms)	−0.33	−1.53 to 0.88	0.60	−2.26	−3.78 to −0.75	<0.001
RMSSD (ln)	−0.02	−0.05 to 0.01	0.14	−0.04	−0.08 to −0.01	0.02
HF (ln)	−0.05	−0.11 to 0.01	0.12	−0.08	−0.15 to 0.00	0.06
LF (ln)	0.02	−0.03 to 0.07	0.37	−0.10	−0.16 to −0.04	<0.001
Adjusted model ^a^ (*n* = 490)						
Heart rate (bpm)	0.02	−0.38 to 0.42	0.91	0.58	0.08 to 1.08	0.02
SDNN (ms)	−0.39	−1.58 to 0.79	0.52	−1.12	−2.62 to 0.39	0.15
RMSSD (ln)	−0.01	−0.04 to 0.01	0.34	−0.01	−0.05 to 0.02	0.44
HF (ln)	−0.03	−0.09 to 0.03	0.37	−0.01	−0.09 to 0.06	0.71
LF (ln)	0.01	−0.04 to 0.06	0.61	−0.06	−0.12 to 0.01	0.07

Note: work and leisure were included in the same model for each outcome. RMSSD: root mean squared successive differences of R-R intervals; SDNN: the standard deviation of R-R intervals; HF: high-frequency power; LF: low-frequency power; ln: natural logarithm. ^a^ Adjusted for age, gender, body mass index, smoking, and moderate-to-vigorous physical activity during leisure.

**Table 4:** Associations between standing time (h/day) and nocturnal resting heart rate and heart rate variability by activity domain. Results from linear regression analyses. Danish Physical activity cohort with objective measurements (DPHACTO), 2013.

	Standing at Work				Standing During Leisure		
	B	95% CI	*p*		B	95% CI	***p***
Unadjusted model (*n* = 490)							
Heart rate (bpm)	−0.65	−1.24 to −0.05	0.03		0.73	−0.23 to 1.70	0.14
SDNN (ms)	1.50	−0.29 to 3.29	0.10		1.70	−1.20 to 4.60	0.25
RMSSD (ln)	0.05	0.00 to 0.09	0.04		0.02	−0.05 to 0.09	0.59
HF (ln)	0.09	0.00 to 0.19	0.05		0.05	−0.09 to 0.20	0.47
LF (ln)	0.05	−0.03 to 0.12	0.21		0.06	−0.06 to 0.18	0.33
Adjusted model ^a^ (*n* = 490)							
Heart rate (bpm)	−0.70	−1.27 to −0.13	0.02		0.49	−0.53 to 1.52	0.34
SDNN (ms)	1.21	−0.50 to 2.91	0.17		2.16	−0.90 to 5.22	0.17
RMSSD (ln)	0.04	0.00 to 0.08	0.05		−0.01	−0.09 to 0.06	0.77
HF (ln)	0.08	−0.01 to 0.16	0.07		−0.02	−0.17 to 0.14	0.84
LF (ln)	0.04	−0.03 to 0.11	0.27		0.11	−0.01 to 0.22	0.08

Note: work and leisure were included in the same model for each outcome. RMSSD: root mean squared successive differences of R-R intervals; SDNN: the standard deviation of R-R intervals; HF: high-frequency power; LF: low-frequency power; ln: natural logarithm; CI: confidence interval. ^a^ Adjusted for age, gender, body mass index, smoking, and moderate-to-vigorous physical activity during leisure.

## Supplementary Materials

The following are available online at https://www.mdpi.com/1660-4601/16/4/650/s1, Table S1: Descriptive data of nocturnal resting heart rate and heart rate variability (*n* = 490). Table S2: Association between standing time (h/day) and nocturnal resting heart rate and heart rate variability, stratified by age. Adjusted model ^a^ (*n* = 490). Table S3: Association between standing time (h/day) and nocturnal resting heart rate and heart rate variability stratified by occurrence of CVD. Adjusted model ^a^ (*n* = 489).
