# Supplementary material for: Objectively Measured Sitting and Standing in Workers: Cross-Sectional Relationship with Autonomic Cardiac Modulation
Source: Int J Environ Res Public Health. 2019 Feb 22;16(4):650. doi: 10.3390/ijerph16040650 (PMC6406480; doi:10.3390/ijerph16040650)
Supplement: Supplementary file 1 [file ijerph-16-00650-s001.pdf]

Table S1. Descriptive data of nocturnal resting heart rate and heart rate variability (n = 490).

	Mean	SD
Heart rate (bpm)	56.8	7.5
SDNN (ms)	55.9	22.5
RMSSD (ln)	3.8	0.6
HF (ln)	6.3	1.2
LF (ln)	6.5	0.9

Abbreviations: RMSSD, root mean squared successive differences between R-R intervals; SDNN, standard deviation of R-R intervals; LF, low frequency power; HF, high frequency power.

Table S2. Association between standing time (h/day) and nocturnal resting heart rate and heart rate variability, stratified by age. Adjusted model[a] (n = 490).

	**<45 years (n = 196)**			**≥45 years (n = 294)**		
	B	95% CI	p	B	95% CI	p
**Standing at work**						
Heart rate (bpm)	−0.86	−1.75 to 0.03	0.06	−0.55	−1.32 to 0.21	0.15
SDNN (ms)	1.76	−1.27 to 4.80	0.25	0.86	−1.20 to 2.93	0.41
RMSSD (ln)	0.05	−0.02 to 0.11	0.17	0.04	−0.02 to 0.09	0.20
HF (ln)	0.09	−0.05 to 0.22	0.21	0.08	−0.03 to 0.19	0.17
LF (ln)	0.08	−0.03 to 0.19	0.14	0.01	−0.08 to 0.10	0.84
**Standing during leisure**						
Heart rate (bpm)	0.61	−0.97 to 2.19	0.45	0.43	−0.93 to 1.78	0.54
SDNN (ms)	−0.22	−5.60 to 5.17	0.94	3.82	0.15 to 7.49	0.04
RMSSD (ln)	−0.07	−0.18 to 0.05	0.27	0.03	−0.07 to 0.12	0.59
HF (ln)	−0.14	−0.38 to 0.11	0.27	0.06	−0.14 to 0.26	0.54
LF (ln)	0.08	−0.11 to 0.26	0.42	0.13	−0.02 to 0.28	0.10

[a] Adjusted for age, gender, body mass index, smoking and moderate-to-vigorous physical activity during leisure. Abbreviations: RMSSD, root mean squared successive differences between R-R intervals; SDNN, standard deviation of R-R intervals; LF, low frequency power; HF, high frequency power.

Table S3. Association between standing time (h/day) and nocturnal resting heart rate and heart rate variability stratified by occurrence of cardiovascular disease (CVD). Adjusted model[a] (n = 489).

	**No CVD (n = 259)**			**CVD (n = 230)**		
	B	95% CI	p	B	95% CI	p
**Standing at work**						
Heart rate (bpm)	−0.54	−1.26 to 0.18	0.14	−0.79	−1.72 to 0.14	0.09
SDNN (ms)	0.27	−2.15 to 2.69	0.83	1.98	−0.48 to 4.44	0.11
RMSSD (ln)	0.03	−0.03 to 0.09	0.29	0.04	−0.02 to 0.10	0.21
HF (ln)	0.07	−0.04 to 0.19	0.22	0.07	−0.07 to 0.20	0.33
LF (ln)	0.02	−0.08 to 0.11	0.74	0.07	−0.04 to 0.17	0.22
**Standing during leisure**						
Heart rate (bpm)	0.74	−0.54 to 2.02	0.26	0.02	−1.68 to 1.71	0.98
SDNN (ms)	0.60	−3.69 to 4.90	0.78	4.11	−0.38 to 8.60	0.07
RMSSD (ln)	−0.08	−0.18 to 0.02	0.11	0.08	−0.03 to 0.20	0.17
HF (ln)	−0.14	−0.35 to 0.06	0.17	0.15	−0.09 to 0.39	0.21
LF (ln)	0.09	−0.06 to 0.25	0.24	0.13	−0.05 to 0.31	0.17

[a] Adjusted for age, gender, body mass index, smoking and moderate-to-vigorous physical activity during leisure. Abbreviations: RMSSD, root mean squared successive differences between R-R intervals; SDNN, standard deviation of R-R intervals; LF, low frequency power; HF, high frequency power.
